# 607. Improving Health Maintenance Among Patients with HIV by Implementing a SmartPhrase and a Care Gap in the EPIC Electronic Medical Record

**DOI:** 10.1093/ofid/ofab466.805

**Published:** 2021-12-04

**Authors:** Yuriko Fukuta, Thomas P Giordano

**Affiliations:** Baylor College of Medicine, Bellaire, Texas

## Abstract

**Background:**

Most deaths in HIV-infected patients receiving antiretroviral therapy are now related to conditions other than AIDS. HIV infection appears to increase the risk of many non-AIDS-related conditions, highlighting the importance of preventive care, however, recommended health maintenance items unique patients with HIV (PWH) are not always accomplished. We aimed to improve health maintenance by implementing a SmartPhrase and a Care Gap package in the EPIC Electronic Medical Record (EMR).

**Methods:**

We developed a HIV health maintenance SmartPhrase in EPIC that included the last screening dates for syphilis, gonorrhea, chlamydia, hepatitis A, hepatitis B, hepatitis C, latent tuberculosis, hyperlipidemia, diabetes and human papilloma virus and the dates of receipt of hepatitis A vaccines, hepatitis B vaccines, pneumococcal conjugate vaccines, pneumococcal polysaccharide vaccines and influenza vaccines (Figure 1). Providers can select their plan for each health maintenance item based on these data and their plans are documented in the encounter notes. Providers were educated to use the SmartPhrase in each office visit. An HIV registry was built after choosing 509 HIV related medical conditions. The health maintenance topics were displayed in a “Care Gaps” summary using the data in the HIV registry (Figure 2). Completion rates for the health maintenance items were compared before and after implementation. The health maintenance package was implemented on 3/1/2020.

Figure 1. SmartPhrase .IDNOTE description and note documentation

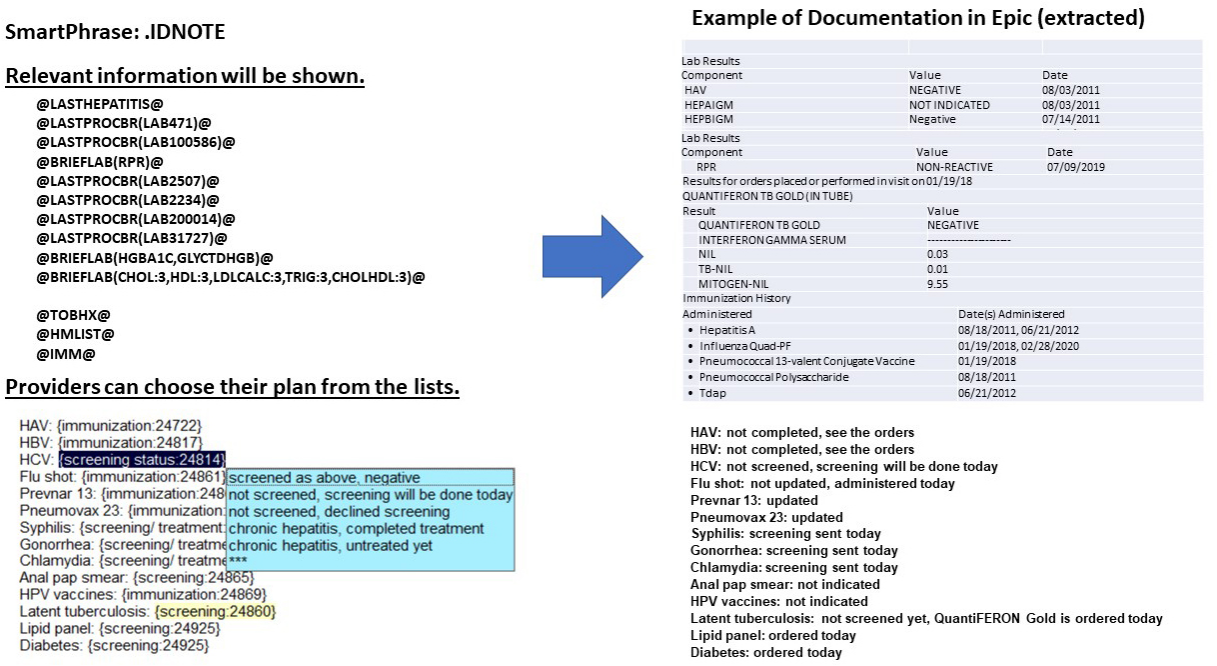

Information relevant to health maintenance and providers' plan for each health maintenance are documented in the encounter notes.

Figure 2. CareGaps© 2021 Epic Systems Corporation

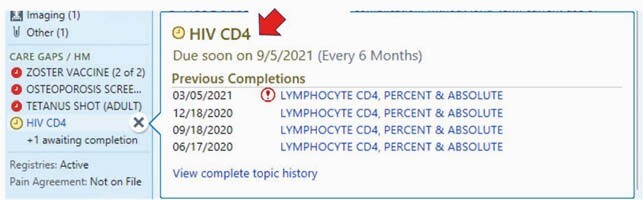

CD4 every 6 months is displayed as a part of the health maintenance in a “Care Gaps” summary using the data in the HIV registry, whether their HIV is well controlled or not.

**Results:**

Of the 380 patients in the registry, 162 had office visits with the ID clinic from 1/1/20 to 6/5/20. Chart review of 100 patients who had office visits after implementation was performed and compared to the 62 patients prior to implementation (Table 1). The rates of hepatitis A vaccination (*P*= 0.001), hepatitis B vaccination (*P=* 0.05) and influenza vaccination (*P=*0.035) were increased significantly. Pneumonia vaccine administrations and anal pap smear performance compliance remained suboptimal. Providers reported that the time they spent searching for lab results and immunization records and documenting were shortened.

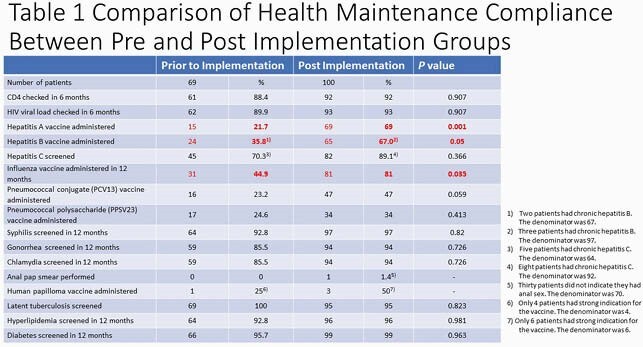

The rates of hepatitis A vaccination (P= 0.001), hepatitis B vaccination (P= 0.05) and influenza vaccination (P=0.035) were increased significantly.

**Conclusion:**

A health maintenance package consisting of a SmartPhrase and summary display in the EMR with provider education likely helps improve health maintenance in PWH.

**Disclosures:**

**All Authors**: No reported disclosures

